# The role of costimulatory molecules in glioma biology and immune microenvironment

**DOI:** 10.3389/fgene.2022.1024922

**Published:** 2022-11-09

**Authors:** Ji Wang, Zi Wang, Wenxue Jia, Wei Gong, Bokai Dong, Zhuangzhuang Wang, Meng Zhou, Chunlei Tian

**Affiliations:** ^1^ Department of Neurosurgery, Yichang Central People’s Hospital, The First College of Clinical Medical Science, Institute of Neurology, China Three Gorges University, Yichang, China; ^2^ Department of Emergency, The First People’s Hospital of Yichang, The People’s Hospital of China Three Gorges University, Yichang, China

**Keywords:** glioma, costimulatory molecule, signature, prognosis, immunotherapy

## Abstract

**Background:** Extensive research showed costimulatory molecules regulate tumor progression. Nevertheless, a small amount of literature has concentrated on the potential prognostic and therapeutic effects of costimulatory molecules in patients with glioma.

**Methods:** The data were downloaded from The Cancer Genome Atlas (TCGA) database, Chinese Glioma Genome Atlas (CGGA) database, and Gene Expression Omnibus (GEO) database for bioinformatics analysis. R software was applied for statistical analysis. Using the FigureYa and Xiantao online tools (https://www.xiantao.love/) for mapping.

**Results:** The Least absolute shrinkage and selection operator (LASSO) and Cox regression analysis were utilized to identify the signature consisting of five costimulatory molecules. Multivariate regression analysis revealed that the prognosis of glioma could be independently predicted by the riskscore. Furthermore, we explored clinical and genomic feature differences between the two groups. The level of tumor mutational burden (TMB) was higher in the high-risk group, while more mutation of IDH1 was observed in the low-risk group. Results of Tumor Immune Dysfunction and Exclusion (TIDE) analysis showed that high-risk patients were more prone to be responded to immunotherapy. In addition, subclass mapping analysis was performed to validate our findings and the results revealed that a significantly higher percentage of immunotherapy response rate was observed in the high-risk group.

**Conclusion:** A novel signature with a good independent predictive capacity of prognosis was successfully identified. And our findings reveal that patients with high-risk scores were more likely to be responded to immunotherapy.

## Introduction

Gliomas are the most common and aggressive primary tumors of brain, with approximately 200,000 deaths worldwide (Sung et al., 2021). Generally, according to the World Health Organization (WHO) standards, grades I-II are low-grade gliomas (LGGs) and grades III-IV are high-grade gliomas (HGGs) (Figarella-Branger et al., 2021). It is worth noting that glioblastoma (GBM) with WHO grade IV, well known for their aggressiveness and high propensity to metastasize, have only a 5% five-year survival rate (Shergalis et al., 2018). Despite major advances in cancer treatment and many emerging therapies that have been proposed for glioma patients, the overall survival rate (OS) has not significantly increased in recent years (Xu et al., 2020). Under most circumstances, patients with glioma have advanced into the intermediate and advanced stages at the time of diagnosis, missing out on the optimal treatment time (Wang et al., 2020a). Currently, WHO grade is most commonly used as a reference to judge the clinical prognosis of glioma patients, which is valuable but insufficient for prognostic prediction and evaluation of subgroups of patients (Ali et al., 2021). Thus, it is important to find additional more effective targets and more susceptible therapeutic options for glioma patients to improve the prognosis.

With the development of molecular biology, immunotherapy has been recognized as a promising treatment for overcoming glioma despite the presence of the blood-brain barrier (BBB) (Ye et al., 2019; Marcucci et al., 2021). Costimulatory molecules and signals consisting of the tumor necrosis factor (TNF) families and B7/CD28 family are promising candidates for immunotherapy (Croft et al., 2013). On the one hand, molecules belonging to B7/CD28 family are essential for triggering immune responses including the most common immune checkpoint inhibitors (ICIs) target PD-1 and PD-L1 (Keir et al., 2008). In another hand, molecules belonging to TNF/TNFR family are essential for the promotion of anti-tumor immunity (Driessens et al., 2009). In recent years, many literatures show that costimulatory molecules are linked to the tumorigenesis and promotion of various cancers. CD40LG (CD40L, TNFSF5, CD154), one of the most well-studied TNFSF, has been a therapy target in cancer treatment and is typically associated with the prognosis of lung cancer (Mu et al., 2015). Overexpression of TNFSF14 can stop or delay the development of human papillomavirus 16-induced tumors *via* enhancing the functional responses of T cells (Kanodia et al., 2010). Moreover, EDAR is an important effector of typical Wnt signaling in the development of skin attachment, which can adjust Wnt/β-catenin signaling pathway to promote the proliferation of colorectal cancer cells (Wang et al., 2020b). Given the prominent values of costimulatory molecules, it is essential to screen the costimulatory molecules associated with the prognosis for improving prognosis evaluations of glioma patients.

To investigate the significant role of costimulatory molecules in glioma, RNA sequencing data was used to systematically analyze the costimulatory molecules expression with distinct clinicopathological features of gliomas in four independent cohorts according to the TCGA and CGGA datasets. A prognostic signature was then developed that could effectively predict the glioma prognosis. Moreover, we investigated discrepancies in clinical and genomic profiles in two risk groups and explored potential targets for therapies.

## Materials and methods

### Data acquisition and processing

The relevant clinical data and RNA-sequencing data of TCGA-LGG patients and TCGA-GBM patients were acquired from the TCGA database (https://portal.gdc.cancer.gov/) as the training cohort. In addition, four datasets including data of glioma were retrieved from the CGGA and GEO databases (https://cgga.org.cn/ and https://www.ncbi.nlm.nih.gov/geo/) as the validation cohorts. The Genotype-Tissue Expression (GTEx) database (https://gtexportal.org/) RNA-Seq data was also downloaded for further analysis. Then, data was merged, converted to TPM values (Zhao et al., 2020), annotated with probes, and removed samples with incomplete information and duplicates. The data were then batch normalized using the “ComBat” algorithm to decrease the possibility of batch effects in disparate datasets. The expression profile of patients who responded to immunotherapies was collected from the known literature and was applied for predicting the immunotherapy response of glioma patients (Roh et al., 2017). Single-sample GSEA analysis (ssGSEA) was conducted *via* the “GSVA” R package (version 4.0.2).

### Differential expression analysis

All costimulatory molecules were gotten from the published literature (Aye et al., 2021). RNA sequencing expression data of 56 costimulatory molecules in our research including normal samples and tumor samples were obtained *via* UCSC XENA data hubs (https://tcga.xenahubs.net). Then differential expression analysis of all these costimulatory molecules was conducted *via* the “EdgeR” R package (version 4.0.2), and we visualized the results *via* the “ggplot2” R package (version 4.0.2).

### Establishment and evaluation of the prognostic feature in a combined glioma cohort

After eliminating patients with survival time less than 30 days and deficient clinic information, 622 patients in the TCGA database were used to establish a prognostic signature, and 929 patients in the CGGA database were utilized to estimate the predictive ability and reliability of the signature. First, univariate Cox regression was conducted to obtain the costimulatory molecules related to OS of glioma patients (*p* < 0.01). The LASSO regression analysis was then conducted *via* the “glmnet” R package (version 4.0.2) to confirm the selected hub genes further. Finally, the prognostic signature was defined *via* a multivariate Cox regression analysis and the riskscore was obtained for each patient using the following formula: Riskscore = coef1*costimulatory molecule + coef2*costimulatory molecule2 + coef3*costimulatory molecule3 + … + coefN*costimulatory molecules. Then, patients with glioma were divided into two groups according to the riskscore.

The OS time and the AUC corresponding to 1‐, 3‐, and 5-years were compared between two risk groups based on the Kaplan‐Meier (KM) survival analysis. Furthermore, univariate and multivariate Cox regression analyses were conducted to explore the independence of the riskscore as a predictor by comparing the riskscore and different clinical factors.

### GSEA and mutation analysis

The “ClusterProfiler” R package (version 4.0.2) was utilized for performing GSEA. Curated gene sets, oncogenic signature gene sets, ontology gene sets and hallmark gene set (https://www.gsea-msigdb.org/gsea/downloads.jsp) was recognized as the reference sets to investigate the discrepancy in the tumor genetic pathways. Immunotherapy-related positive signatures from the known literature were obtained to conduct the correlation analysis with riskscore (Hu et al., 2021). Moreover, somatic mutation data used to calculate tumor mutational burden (TMB) were available from the cBioPortal website (https://www.cbioportal.org/datasets). Differentially mutated genes with *p*-value < 0.05 in two risk groups were screened and maftools were applied for analyzing the interaction between gene mutations.

### Investigation of immune signatures and immunotherapeutic response prediction

The scores of ESTIMATE, immune, and stromal were counted by “estimate” R package (version 4.0.2). The ssGSEA algorithm was applied for quantifying the enrichment scores of 39 immune signatures. The 48 immune-checkpoint-relevant genes expression were selected for disparate expression analysis in two risk groups. TIDE analysis could precisely model immune escape and predict cancer response to immunotherapy (Jiang et al., 2018). Patients with TIDE score >0 were considered to have no immunotherapy response, and patients with TIDE score <0 were considered to have the immunotherapy response. Moreover, a subclass mapping algorithm (https://cloud.genepattern.org/gp), was utilized for determining which group was more likely to benefit from immunotherapy (Hoshida et al., 2007). In addition, appropriate targeted drugs were defined by differential expression analysis of drug sensitivity.

### Statistical analysis

According to the R software (version 4.0.2), all analyses were conducted. All statistical tests were two-sided, and the difference was statistically significant when *p*-value <0.05. Continuous variables with normal distribution were contrasted *via* an independent *t*-test. Wilcoxon rank-sum test was applied for comparing continuous variables.

## Results

### Data combination and correction for batch effect

**FIGURE 1:**
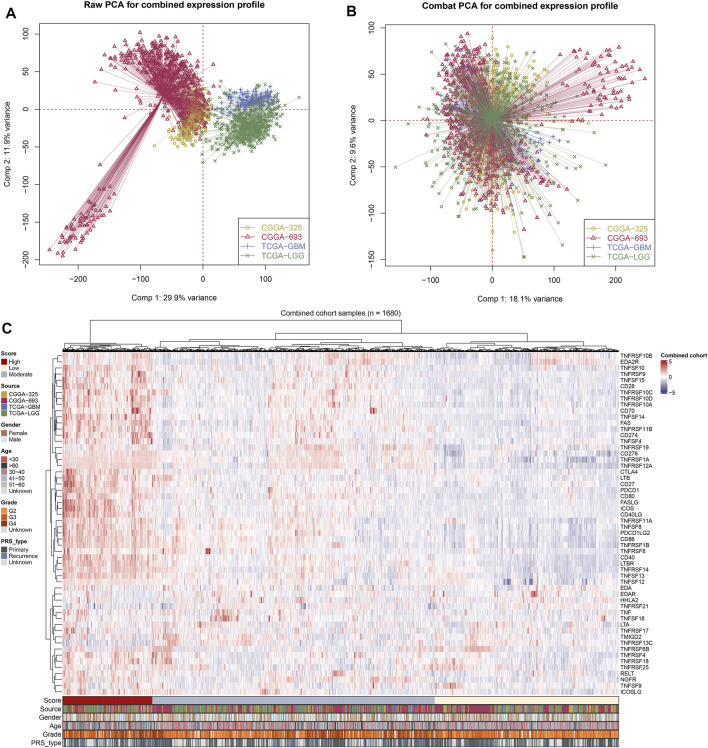
Integration of glioma cohorts and the expression of costimulatory molecules. **(A)** Used in our analysis of the four glioma cohorts had significant batch differences (Comp 1: 29.9% variance, Comp 2: 11.9% variance). **(B)** The “sva” R package for glioma cohort combinations significantly decreased the batch difference (Comp 1: 18.1% variance, Comp 2: 9.6% variance). **(C)** Costimulatory molecules expression profile of all patients.

After comprehensively retrieving the TCGA and CGGA databases, four glioma cohorts conformed to our standard, including TCGA-GBM, TCGA-LGG, CGGA_325, and CGGA_693 databases. Meanwhile, we found an evident batch effect in the four datasets (Figure 1A). The “sva” R package was applied for eliminating the potential batch effect, and inter-assay differences were found significantly decreased in the conjunct glioma cohort (Figure 1B). Moreover, the expression of costimulatory molecules in each patient was quantified *via* the ssGSEA algorithm for further analysis (Figure 1C).

### Costimulatory molecules-based prognosis signature

**FIGURE 2:**
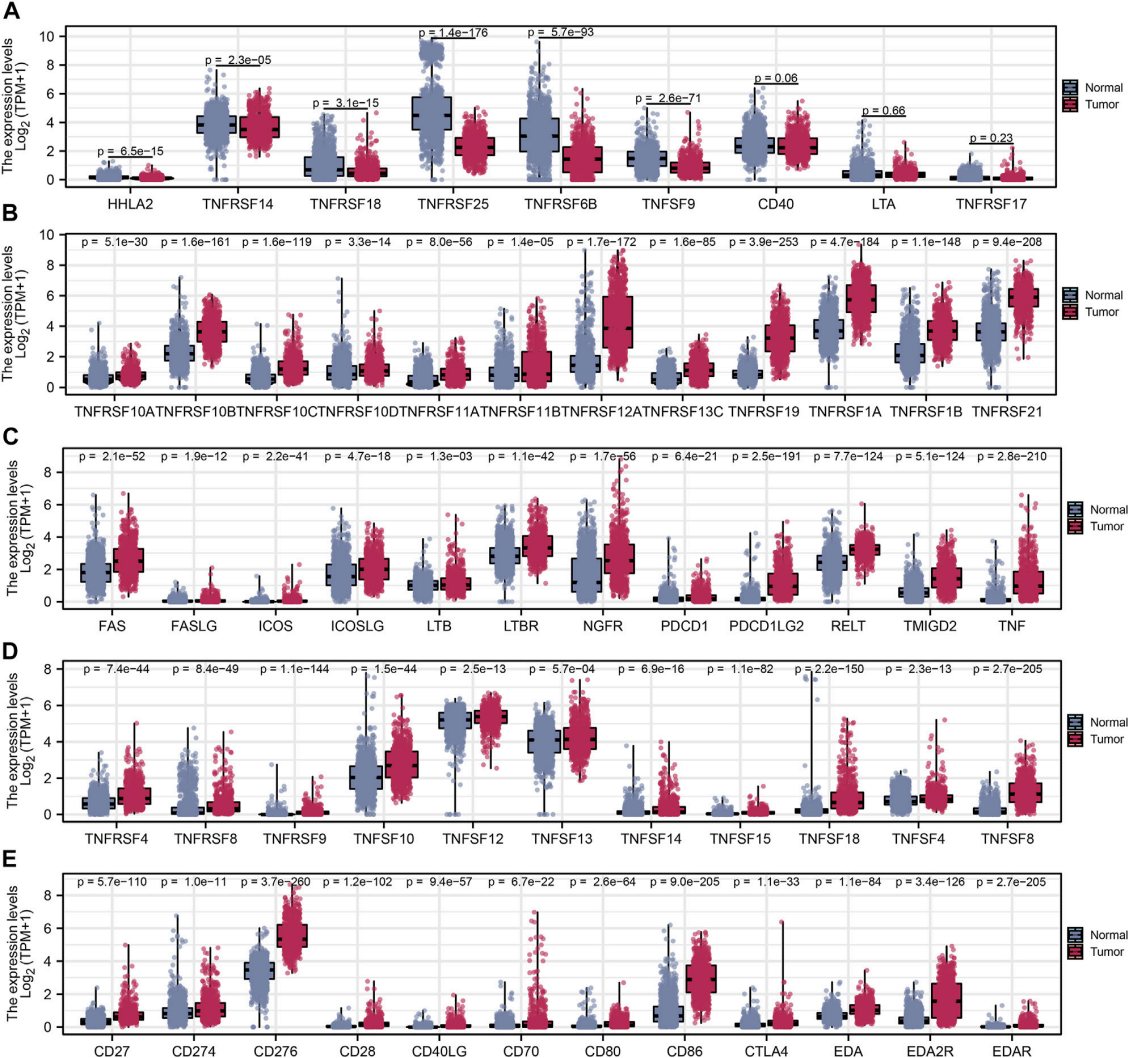
Differential expression analysis of costimulatory molecules. **(A)** Six costimulatory molecules HHLA2, TNFRSF14, TNFRSF18, TNFRSF25, TNFRSF6B, and TNFSF9 were underexpressed in tumor samples. **(B–E)** The other 48 costimulatory molecules were highly expressed in tumor samples.

**FIGURE 3:**
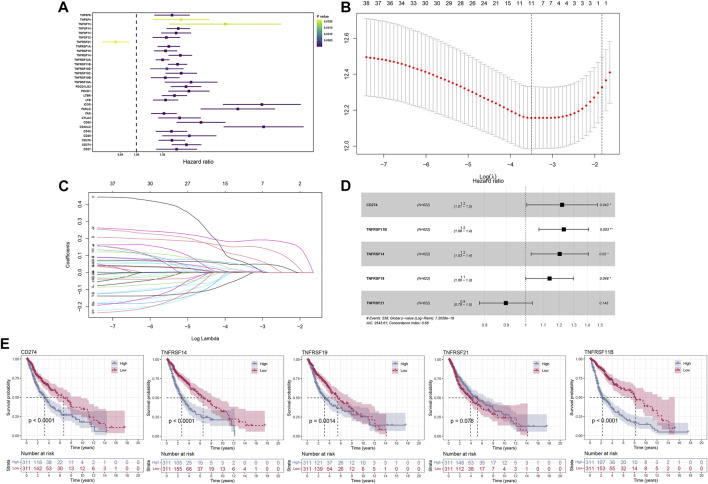
The construction of the model based on costimulatory molecules. **(A)** 31 costimulatory molecules, including 1 protective factor and 30 risk factors, were identified by univariate regression analysis. **(B,C)** LASSO regression analysis was performed on costimulatory molecules identified by univariate Cox regression analysis. **(D)** Multivariate Cox regression analysis identified five costimulatory molecules for model construction. **(E)** KM survival curves analysis was performed on five model costimulatory molecules.

After a comprehensive search performed on public databases, 1,152 normal samples from the GTEx database, 689 tumor samples, and five adjacent samples from the TCGA database were screened. Differential expression analyses of all costimulatory molecules were performed between normal samples from TCGA and GTEx databases, and tumor samples from the TCGA database. 57 differentially expressed costimulatory molecules with *p* < 0.001 were identified, among which the expression of six costimulatory molecules including HHLA2, TNFRSF14, TNFRSF18, TNFRSF25, TNFRSF6B, and TNFSF9 were decreased in tumor samples (Figure 2A), whereas other costimulatory molecules were increased in tumor samples (Figures 2B–E). Patients with intact survival information were screened for further analysis (Supplementary Table S1). The training cohort was the TCGA cohort and the verification cohort was the CGGA cohort. Univariate Cox regression analysis was performed on the differentially expressed costimulatory molecules and 31 costimulatory molecules were screened (Figure 3A). Next, 11 OS-related costimulatory molecules were identified by LASSO Cox regression analysis (Figures 3B,C). According to five costimulatory molecules defined by multivariate Cox regression analysis, a prognosis signature was constructed and the riskscore was calculated by the formula: riskscore = CD274 * 0.198666431 + TNFRSF11B * 0.207922941 + TNFRSF14 * 0.18558208 + TNFRSF19 * 0.130431237404669 + TNFRSF21 * −0.1078322 (Figure 3D). Then, KM survival analysis of these five costimulatory molecules was conducted to contrast the OS time between two groups (Figure 3E).

### Validation of the prognostic feature

**FIGURE 4:**
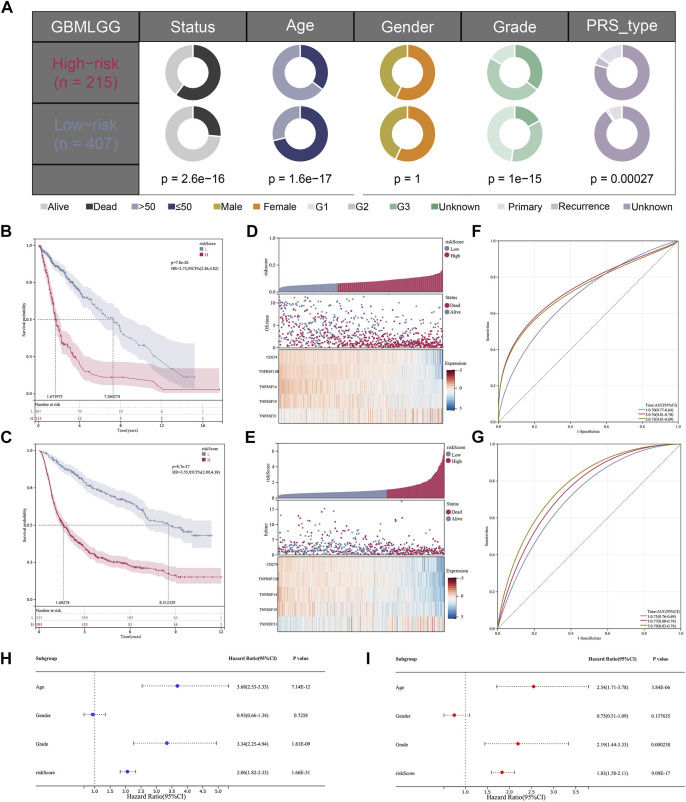
The validation of the model based on costimulatory molecules. **(A)** Clinical correlation analysis of the model. **(B,C)** KM survival curve analysis of the model [**(B)**; HR = 3.72, *p* = 7.8e-26] [**(C)**; HR = 3.55, *p* = 8.7e-37]. **(D,E)** The risk graph shows that the mortality rate of patients increases gradually as their risk value increases. **(F,G)** 1, 3, and 5 years ROC curve analysis of the model. **(H,I)** Univariate and multivariate independent prognostic analysis for the RiskScore.

The optimal truncation value of riskscore 1.07792 in the training cohort and 0.159026 in the validation cohort were counted *via* the “maxstat” R package. Depended on the optimal truncation value, patients were classified into high- and low-risk groups. Clinical information analysis indicated that high-risk patients may have adverse events (Figure 4A). KM survival analysis suggested that in the training cohort (Figure 4B) and validation cohort (Figure 4C), the OS time was markedly shorter in the high-risk group, showing that riskscore could predict the prognosis. As the riskscore increased, there was also a prominent increase in mortality in both the training cohort (Figure 4D) and validation cohort (Figure 4E). Consistently, the AUC value of 1‐, 3‐, and 5 years were respectively 0.70, 0.76, and 0.75 in the training cohort (Figure 4F) and 0.73, 0.77, and 0.79 in the validation cohort (Figure 4G). Moreover, to verify results in microarray datasets, GSE4290 and GSE108474 series were selected to perform the differential expression analysis and the clinical analysis, from which we found the expression of the signature costimulatory molecules was significantly different between tumor and normal tissues (Supplementary Figures S1A,B), and patients with advanced gliomas have a higher riskscore (Supplementary Figures S1C,D). Furthermore, given that the difference between two risk groups may just cause by natural difference between HGG and LGG, we conducted KM and ROC analysis in GBM and LGG patients independently, which were matching our results (Supplementary Figures S2A–F). Univariate and multivariate Cox regression analyses were conducted to demonstrate the possibility of the riskscore being a prognostic element distinct from common clinical factors. With the lowest *p*-value both in univariate and multivariate Cox regression analyses, the riskscore was linked to the prognosis (Figures 4H,I).

### Enrichment analysis and tumor mutation burden

**FIGURE 5:**
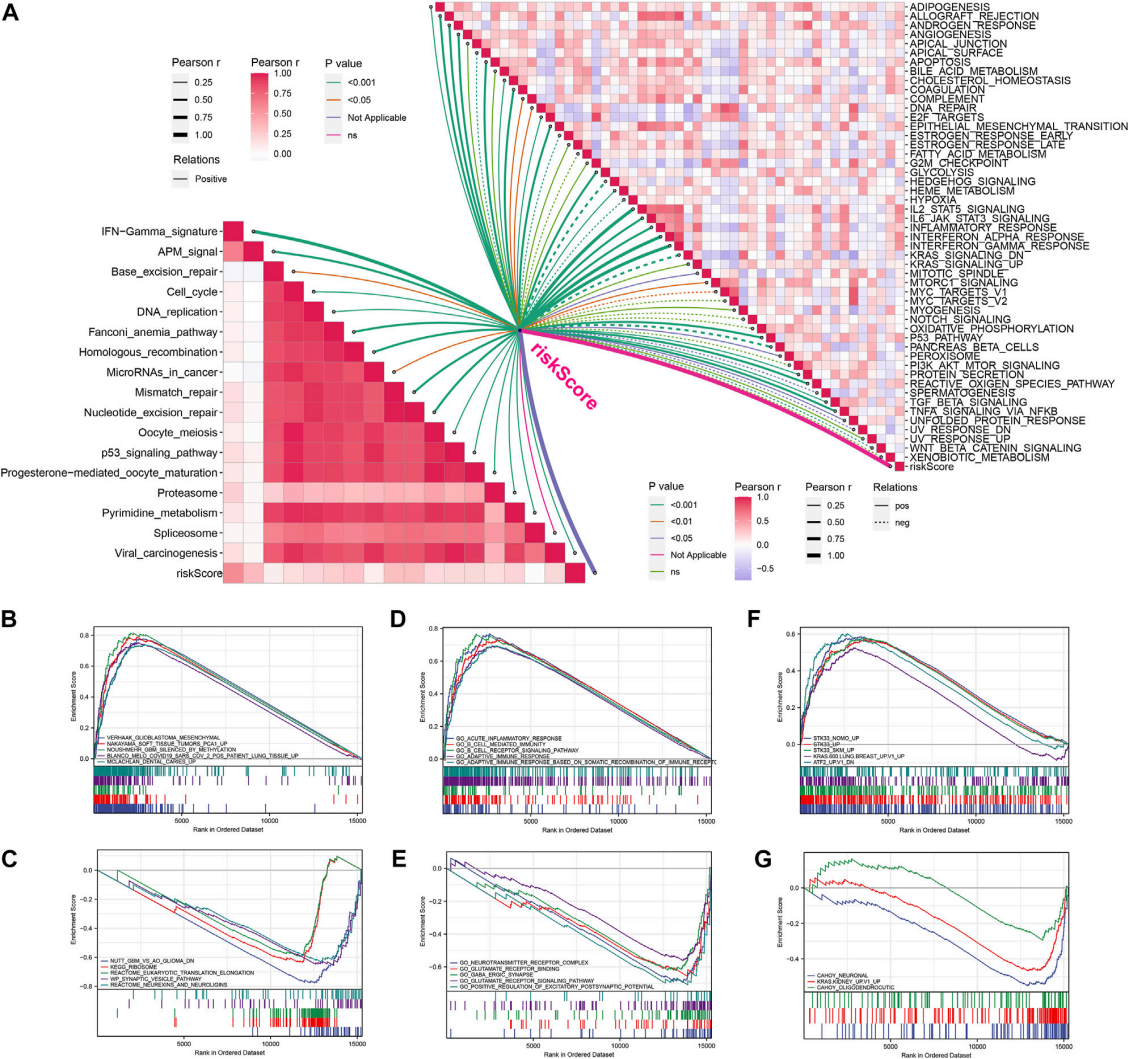
Enrichment analysis of signal pathways. **(A)** Correlations between riskcore and enrichment score of immunotherapy prediction pathway and hallmark gene set. **(B,C)** GSEA analysis between high-risk **(B)** and low-risk **(C)** groups based on curated gene set. **(D,E)** GSEA analysis between high-risk **(D)** and low-risk **(E)** groups based on Ontology gene set. **(F,G)** GSEA analysis between high-risk **(F)** and low-risk **(G)** groups based on oncogenic signature gene set.

**FIGURE 6:**
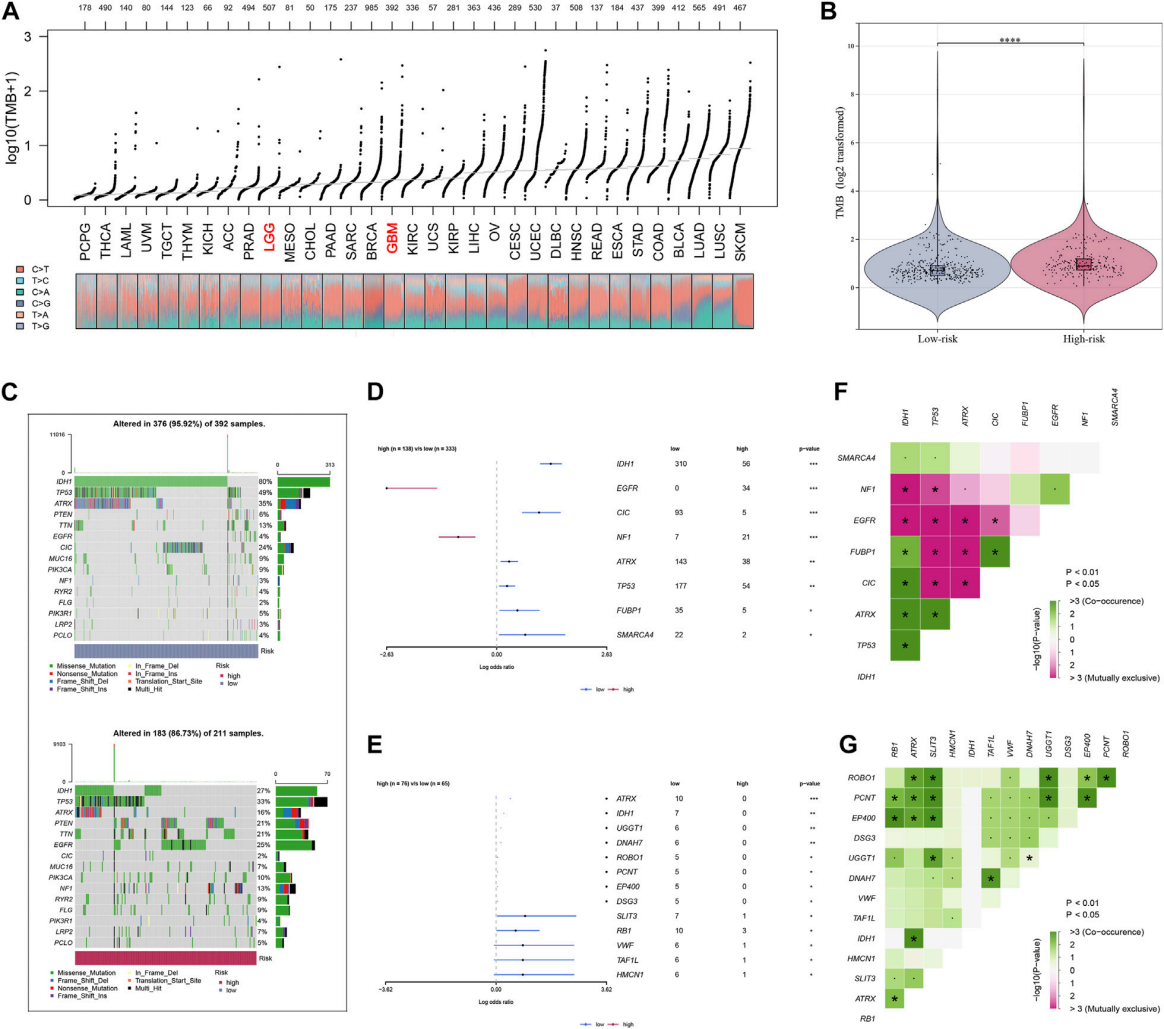
Analysis of tumor mutation characteristics. **(A)** TMB value display of all cancer types in TCGA. **(B)** TMB difference analysis between high- and low-risk groups of glioma patients. **(C)** The profiles of mutations between high- and low-risk groups of glioma patients. **(D,E)** Analysis of mutated gene differences between high- and low-risk groups in patients with glioma. **(F,G)** Co-mutation correlation of mutated genes.

The enrichment scores of known immunotherapy-related positive signatures were calculated for each patient and we found that riskscore positively correlated with all these signatures (Figure 5A), indicating that high-risk patients could be more suitable for anti-tumor immunotherapy. Furthermore, we also analyzed the correlations between riskscore and the scores of the hallmark gene set (Figure 5A). To better understand the biological processes and pathways of riskscore on the patient prognosis in two risk groups, oncogenic signature gene sets, ontology gene sets and curated gene sets were selected to perform GSEA analysis both in high- and low-risk groups patients (Figures 5B–G). Figure 6A showed the TMB level of all cancer types in the TCGA database, from which a clear difference in TMB value was observed between LGG and GBM patients, and the TMB level in high-risk patients was evidently higher than that in low-risk patients (Figure 6B). The somatic mutation profiles revealed that IDH1 mutations were more frequent in low-risk patients and high-risk patients possessed specific top mutated genes (Figure 6C). Given the difference in TMB value between LGG and GBM patients, we then performed differential mutation analysis between two risk groups both in LGG and GBM patients. IDH1, EGFR, CIC, NF1, ATRX, TP53, FUBP1, and SMARCA4 in LGG patients, and ATRX, IDH1, UGGT1, DNAH7, ROBO1, PCNT, EP400, DSG3, SLIT3, RB1, VWF, TAF1L, and HMCN1 in GBM patients were identified that mutated significantly different between two risk groups (Figures 6D,E). Moreover, co-occurrences were confirmed in the mutations of these genes (Figures 6F,G).

### Immunity and immunotherapy response analysis

**FIGURE 7:**
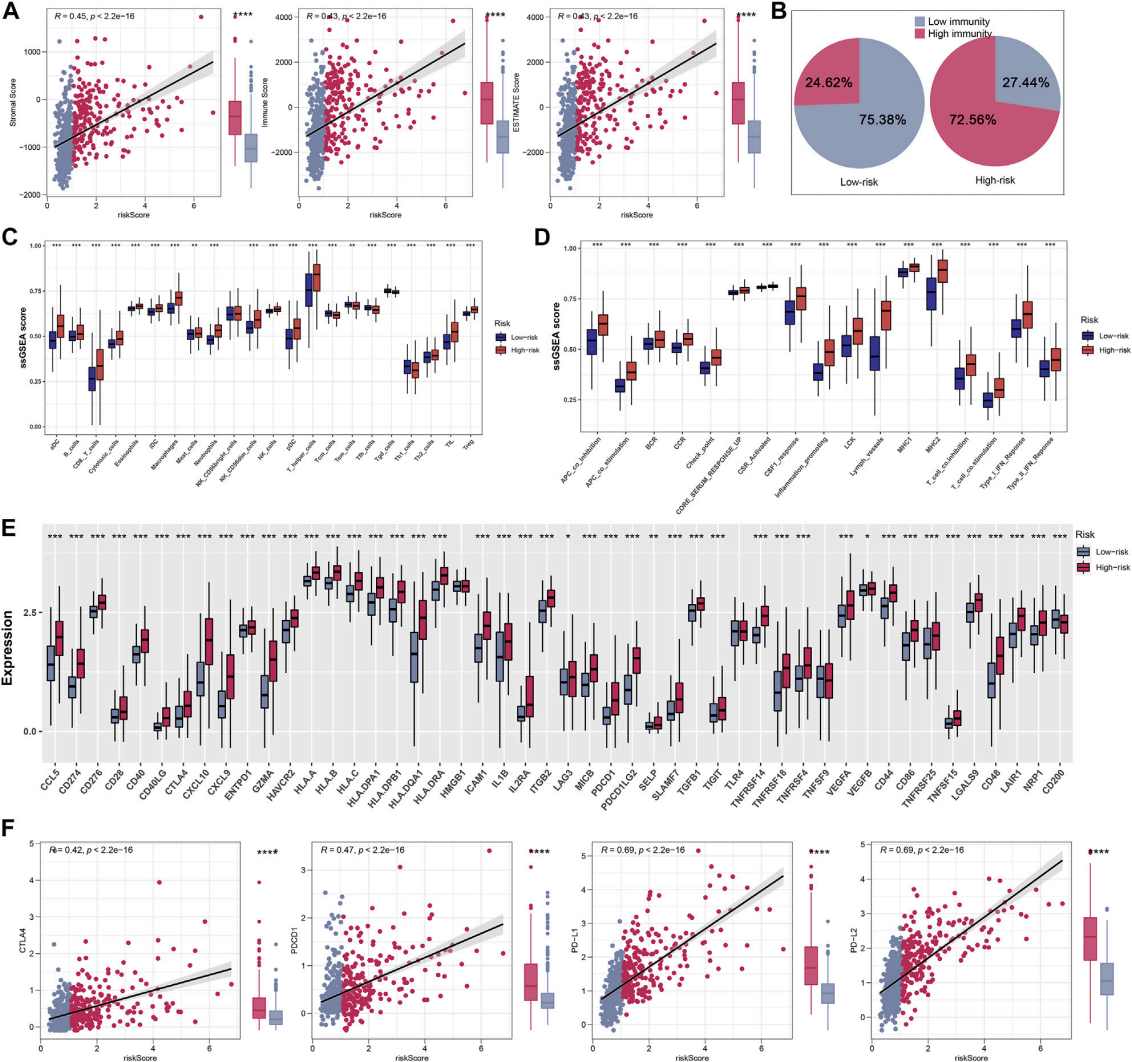
Explore differences in immune characteristics between high- and low-risk groups. **(A)** Differences between the three scores in the high- and low-risk groups. **(B)** Based on 39 immune characteristics, the high-risk group had more highly immunized patients. **(C,D)** Expression differences of 39 immune characteristics between high- and low-risk groups of patients with glioma. **(E)** Differences in expression of common immune checkpoints between high- and low-risk groups of glioma patients. **(F)** Correlation analysis of four immune checkpoints between high- and low-risk groups of glioma patients.

**FIGURE 8:**
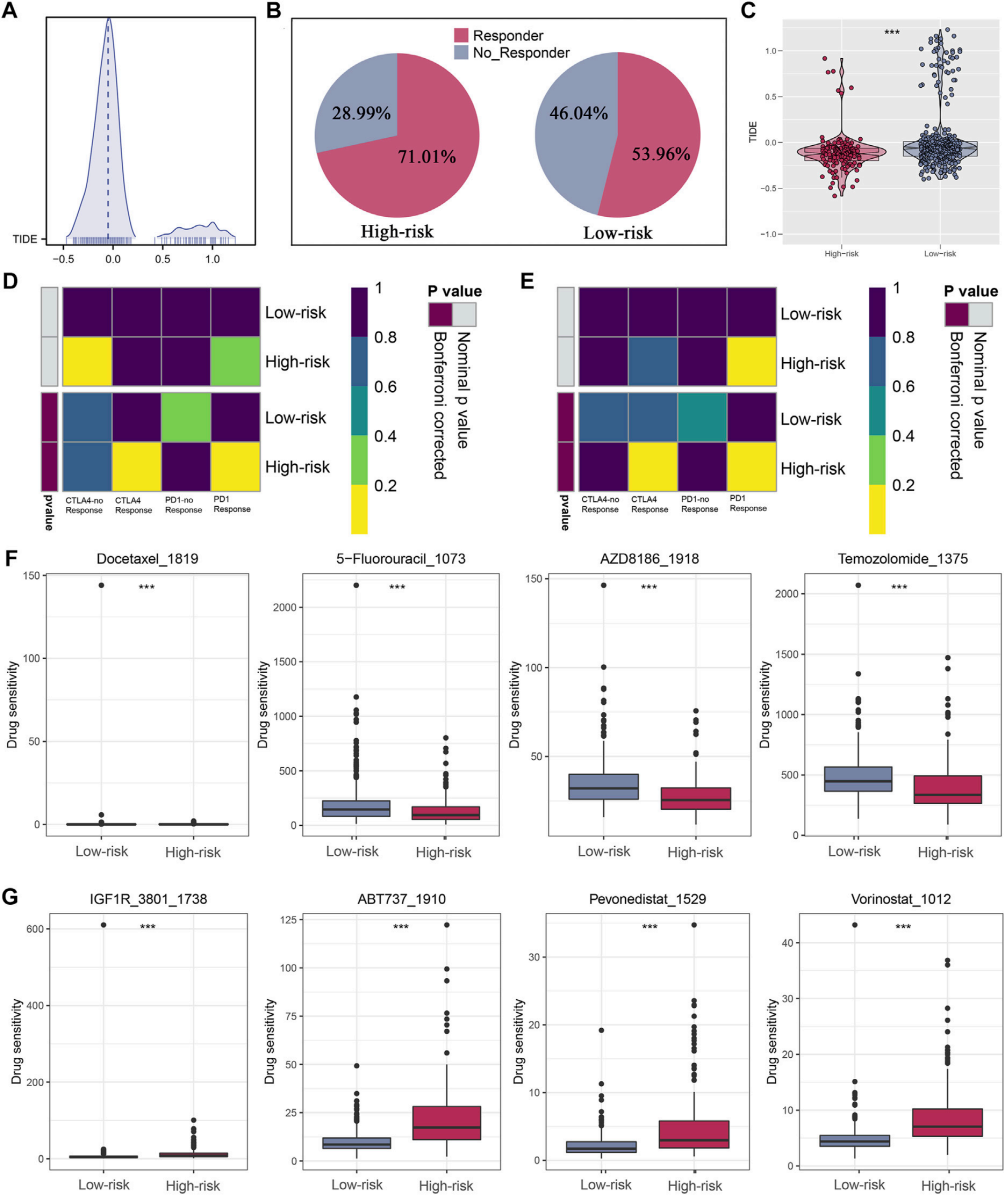
Immunotherapeutic response prediction and screening of potential drugs. **(A)** Distribution of TIDE score. **(B)** Patients in the high-risk group have a higher percentage of responders. **(C)** Patients in the high-risk group have a lower level of TIDE score. **(D,E)** Subclass mapping analysis indicated patients in the high-risk group could be more sensitive to the PD-1 inhibitor in both LGG and GBM. **(F,G)** Drugs with the most significant sensitivity differences in high- and low-risk groups.

The ESTIMATE algorithm was firstly conducted to quantify the immune scores, ESTIMATE scores, and stromal scores, and all the scores were higher in high-riskscore patients (Figure 7A). Then, according to the ssGSEA Z-scores of 39 immune signatures, glioma patients were segmented into high- and low-immunity groups. We could see that high-risk patients consisted of more proportions of the high-immunity group (Figure 7B). Between the two risk groups, there were evident disparities in 39 immune signatures including immune functions and immune cells (Figures 7C,D). In light of the significant role of ICIs therapy in cancer, the expression distribution of 48 immune-checkpoint-correlated genes was presented in Figure 7E. Further, the correlation analysis showed that riskscore had a strong association with PD-1, PD-L1, PD-L2, and CTLA4 expression (Figure 7F). Most notably, all these findings revealed that high-riskscore patients had a stronger immune-signature infiltration. Figure 8A showed the distribution of the TIDE score. In the high-risk group, there were 71.01% of patients responded to immunotherapy, while in the low-risk group, only 53.96% of patients responded to immunotherapy (Figure 8B). Following, we conducted a TIDE analysis of our combined cohort, and the result revealed that the high-risk score linked to the low-TIDE score, demonstrating that these patients may be more likely to respond to immunotherapy (Figure 8C). Moreover, the TIDE analysis in GBM and LGG patients showed the same results independently (Supplementary Figures S3A,B). Besides the TIDE prediction, subclass mapping analysis was applied for verifying our findings, and we were delighted to see that high-riskscore patients were more potential to profit from PD-1 checkpoint treatment both in LGG and GBM patients (Figures 8D,E). Finally, the IC50 for each sample of 179 drugs were estimated *via* R “oncoPredict” package (version 4.0.2) in the GDSC database and between the two risk groups, drugs that have disparities in sensitivity were identified. The drugs with the most prominent sensitivity discrepancies in the two risk groups were displayed in Figures 8F,G.

## Discussion

With the most pernicious kind of primary brain cancer, glioma is famous for its high possibility of metastasis and recurrence (Yang et al., 2022). In the one hand, most glioma patients have developed to the intermediate and advanced stages at the time of diagnosis, missing the optimal treatment time. In another hand, glioma patients lack targeted therapy for specific subtypes and the current treatment can not control glioma invasion to normal adjacent brain tissue (Lee et al., 2018). The prognosis for patients with glioma remains dismal due to intratumoral heterogeneity (Jan et al., 2010). Accumulating evidence revealed a prominent association between different molecular subtypes and clinical outcomes in glioma patients (Li et al., 2022; Zheng et al., 2022). And several studies showed that costimulatory molecules took a great part in initiating anti-tumor immune responses (Zhang et al., 2020), but there was little literature focused on the correlation between costimulatory molecules and stratification of patients with glioma. This study discovered a novel signature that could effectively reflect the survival and immunotherapy response of glioma patients.

In our study, we firstly combined four independent cohorts into a large glioma cohort and used “sva” R package to decrease bias resulting in the small sample size. Univariate Cox analysis verified 31 costimulatory molecules closely correlated to patients’ prognosis and five costimulatory molecules including CD274, TNFRSF11B, TNFRSF14, TNFRSF19, and TNFRSF21 were selected by multivariate Cox analysis for signature construction. Recent research revealed that the expression of CD274 induced under hypoxia condition was signally associated with poor survival in glioma patients (Ding et al., 2021). In addition, TNFRSF11B might be involved in the malignant progression of gliomas and was one of the signature genes that predicted patient prognosis (Kang et al., 2021). TNFRSF14 served a tumor suppressive role by suppressing tumor cell proliferation and inducing apoptosis in bladder cancer and could act as a new diagnosis and prognostic biomarker for bladder cancer (Zhu and Lu, 2018). Further, TNFRSF19, essential for cell proliferation and development of nasopharyngeal carcinoma, represented a mechanism for tumor cells to escape from TGF-β growth-inhibitory action (Deng et al., 2018). Besides, a study demonstrated that the TNFRSF21 expression was strongly negatively linked to the miR-20a-5p expression, and the downregulation of TNFRSF21 functioned as an oncogene in squamous cell carcinoma of the head and neck (Wu et al., 2018). With the in-depth study of costimulatory molecules, the significant costimulatory molecules verified in this study might provide a foundation for further exploring the prognostic and therapeutic role of glioma.

We further investigated the potential biological discrepancy between the two risk groups through GSEA and mutation burden analysis. We found that riskscore positively associated with all known immunotherapy correlated positive signals. With the curated gene sets being a reference set, GSEA analysis indicated that Glioblastoma_Mesenchymal, Nakayama_Soft_Tissue_Tumors_ PCA1_Up, Noushmehr_GBM_Silenced_by_Methylayion, Blanco_ Melo_COVID-19_Sars, Mclachlan_Dental_Caries_Up pathways were evidently stimulated in high-risk patients. And Acute_ Inflammatory_Response, B_cell_Receptor_Signaling_Pathyway, B_Cell_Mediated_immunity, Adaptive_Immune_Response_Based_ On_Somatic_Recombination and Adaptive_Immune_Response were also significantly activated in high-risk patients with the ontology gene sets being a reference set. These findings might partially explain the worse prognosis of high-risk patients and suggested that these patients might be more suitable for anti-tumor immunotherapy. In addition, we found that the TMB level was evidently higher in high-risk patients. Besides, IDH1, associated with a good prognosis in glioma patients, was also observed in more mutations in low-risk patients (Bai et al., 2016).

Moreover, we explored the situation of immune cells and related immune pathways. Results suggested that high-riskscore patients got a higher level of most immune features. We also observed higher levels of PD-L1 and PD-L2 in high-risk patients. Studies had demonstrated that the PD-L1 was a predictive marker for tumor immunotherapy (Patel and Kurzrock, 2015). A study also reported that PD1 expression increased neuronal killing of cancer cells and was associated with long-term survival (Kingwell, 2013). The results of TIDE analysis suggested that in the high-risk group, patients had a higher immunotherapy response rate, which might be correlated to higher immune-checkpoint-related gene levels in high-risk patients. In addition, the subclass mapping algorithm analysis verified that patients with high-risk score were more potential to benefit from PD-1 checkpoint therapy.

To sum it up, this study successfully constructed and validated costimulatory molecules based on the prognostic feature, which might be applied for further guiding treatment and improving clinical outcomes for glioma patients. However, there are several limitations to the study. Firstly, bacause only individuals from Western and China populations are included, the samples may generate some population and genome bias. Then, this study lacks verification from other clinical data sets which will be beneficial to our signature. Finally, given the incompleteness of patient information and the sensitivity to incorrect model specification in our multivariate regression analysis, further analysis of patients with complete clinical information can be very beneficial in the future. Therefore, further investigation of prospective studies and other costimulatory molecules are needed such as functional experiments and underlying molecular mechanisms.

## Conclusion

In conclusion, we investigated the biological features and prognostic value of costimulatory molecules in patients with glioma. We developed a new prognostic signature, and demonstrated the potential immune-related mechanisms of this signature. Then, most importantly, our findings indicated that high-riskscore patients were more likely to benefit from immunotherapy.

## Data Availability

The datasets presented in this study can be found in online repositories. The names of the repository/repositories and accession number(s) can be found in the article/Supplementary Material.

## Supplementary material

SUPPLEMENTARY FIGURE S1 Differential expression analyses and the clinical analyses in microarray datasets. **(A,B)** Results of differential expression analyses showed that patients with glioma had in a higher level of the signature genes in tumor tissues both in GSE4290 **(A)** and GSE108474 series **(B)**. And patients with advanced stage qqhad a higher level of riskscore **(C,D)**.

SUPPLEMENTARY FIGURE S2 The validation of the model using LGG and GBM data independently. **(A,B)** KM survival curve analysis of the model. **(C,D)** The plots shows that the mortality rate of patients increases gradually as their riskscore increases. **(E,F)** 1, 3, and 5 years ROC curve analysis of the model in both LGG and GBM data independently.

SUPPLEMENTARY FIGURE S3 The results of TIDE analyses in LGG and GBM patients independently. **(A,B)** The TIDE analyses showed that patients in high risk group with LGG or GBM all had a lower TIDE score.

## Abbreviations

TCGA, The Cancer Genome Atlas database; CGGA, Chinese Glioma Genome Atlas; TMB, Tumor mutational burden; TIDE, Tumor Immune Dysfunction and Exclusion; WHO, World Health Organization; HGGs, High-grade gliomas; GBM, Glioblastoma; BBB, Blood-brain barrier; TNF, Tumor necrosis factor; ICIs, Immune checkpoint inhibitors; PD-1, Programmed cell death protein 1; PD-L1, Programmed cell death 1 ligand 1; GTEx, Genotype-Tissue Expression; ssGSEA, Single-sample GSEA analysis; KM, Kaplan‐Meier; ROC, Receiver operating characteristics; AUC, Area u Gene set enrichment analysis; LASSO, Least absolute shrinkage and selection operator.
