# Supplementary figures and images for: The role of costimulatory molecules in glioma biology and immune microenvironment
Source: Front Genet. 2022 Nov 9;13:1024922. doi: 10.3389/fgene.2022.1024922 (PMC9682268; doi:10.3389/fgene.2022.1024922)

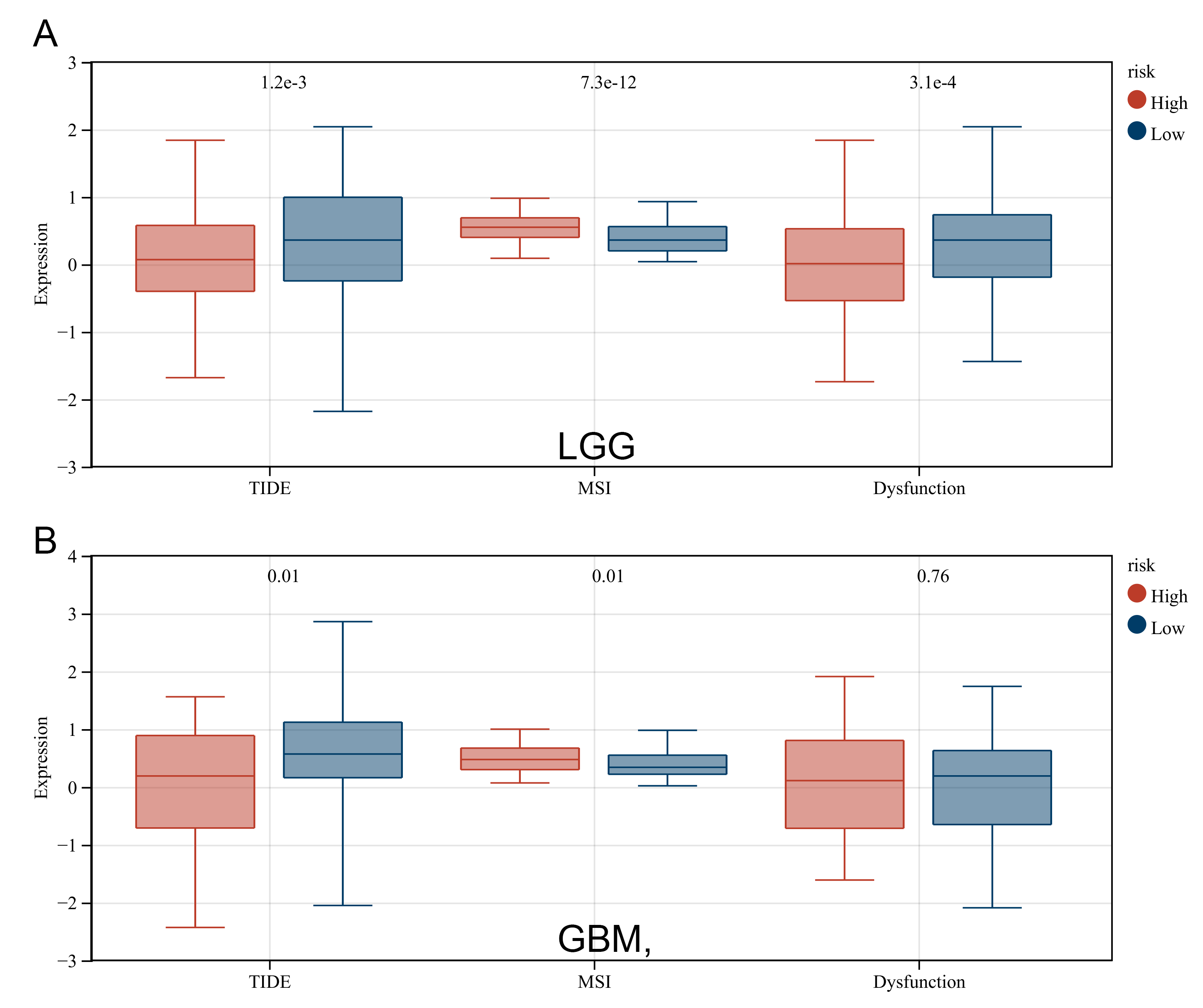

Supplement: Supplementary file 1 [file Image3.TIF]

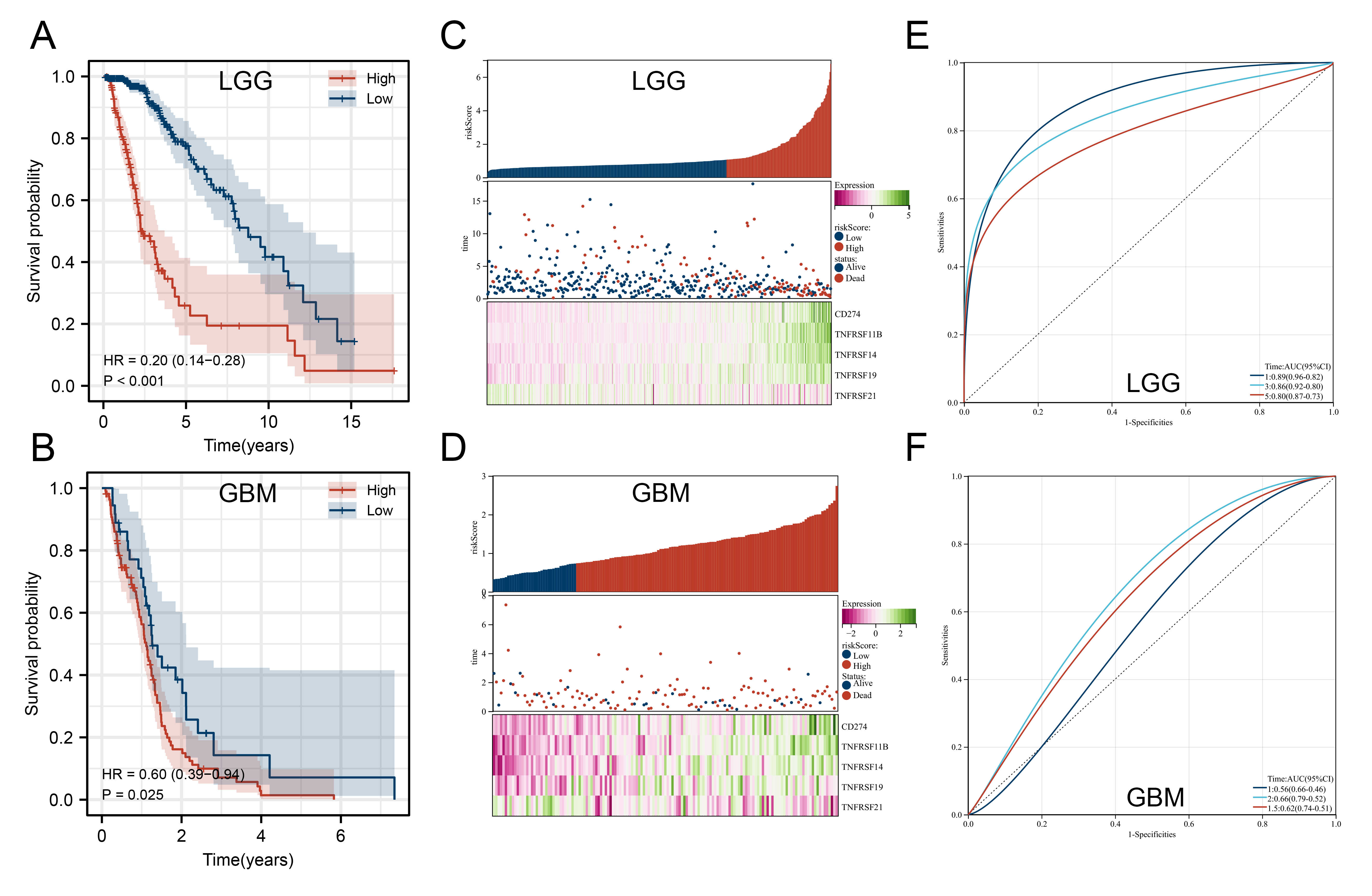

Supplement: Supplementary file 2 [file Image2.TIF]

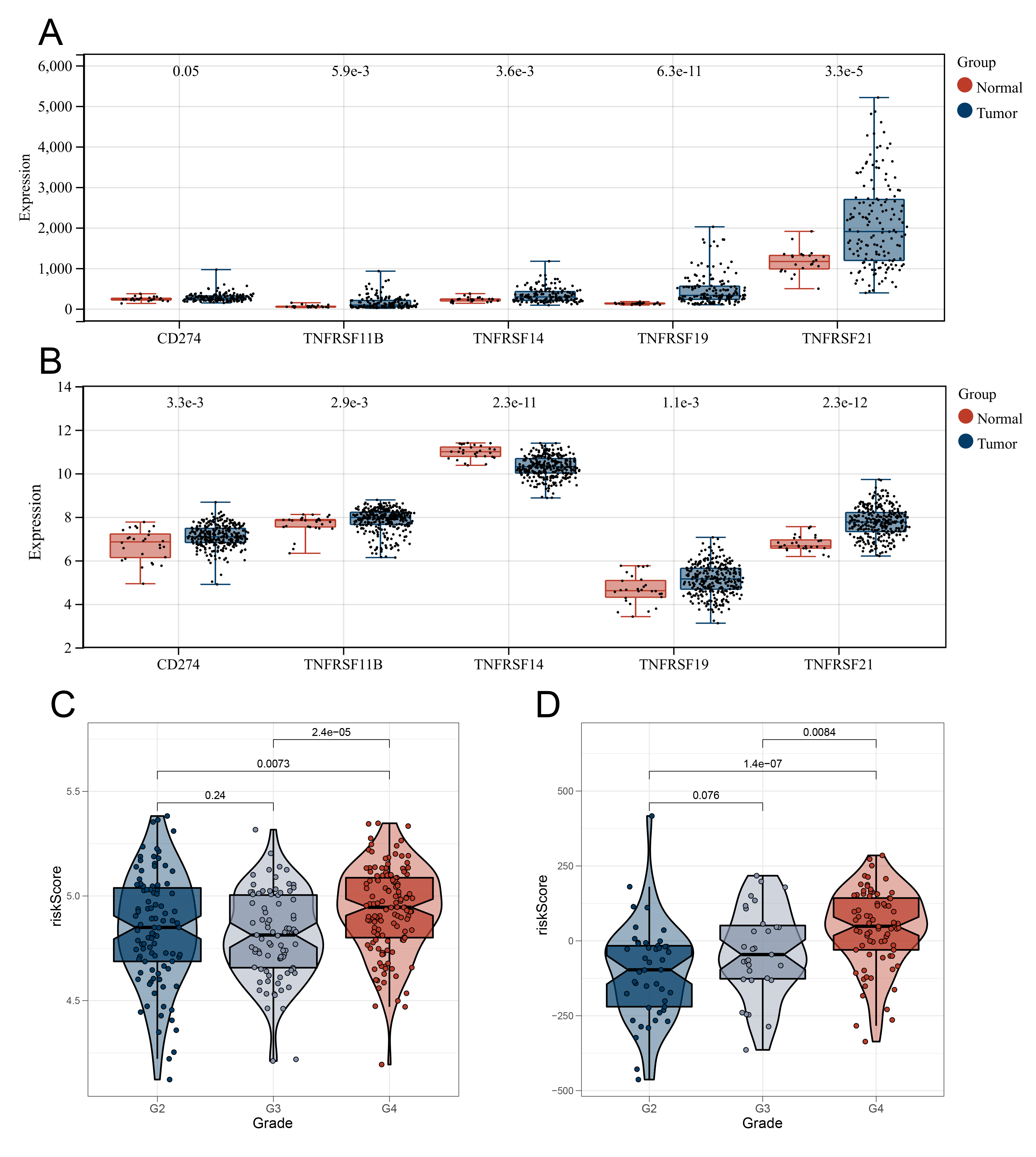

Supplement: Supplementary file 3 [file Image1.TIF]
